# Characterization of H3K9me3 and DNA methylation co-marked CpG-rich regions during mouse development

**DOI:** 10.1186/s12864-023-09758-8

**Published:** 2023-11-03

**Authors:** Hui Yang, Yiman Wang, Yong Zhang

**Affiliations:** Institute for Regenerative Medicine, Department of Neurosurgery, Shanghai East Hospital, Shanghai Key Laboratory of Signaling and Disease Research, Frontier Science Center for Stem Cell Research, School of Life Sciences and Technology, Tongji University, Shanghai, 200092 China

**Keywords:** H3K9me3, DNA methylation, CpG-rich regions, Mouse development, Epigenetic regulation

## Abstract

**Background:**

H3K9me3 and DNA methylation co-marked CpG-rich regions (CHMs) are functionally important in mouse pre-implantation embryos, but their characteristics in other biological processes are still largely unknown.

**Results:**

In this study, we performed a comprehensive analysis to characterize CHMs during 6 mouse developmental processes, identifying over 2,600 CHMs exhibiting stable co-mark of H3K9me3 and DNA methylation patterns at CpG-rich regions. We revealed the distinctive features of CHMs, including elevated H3K9me3 signals and a significant presence in euchromatin and the potential role in silencing younger long terminal repeats (LTRs), especially in some ERVK subfamilies. The results highlight the distinct nature of universal CHMs compared to CpG-rich nonCHMs in terms of location, LTR enrichment, and DNA sequence features, enhancing our understanding of CpG-rich regions' regulatory roles.

**Conclusions:**

This study characterizes the features of CHMs in multiple developmental processes and broadens our understanding of the regulatory roles of CpG-rich regions.

**Supplementary Information:**

The online version contains supplementary material available at 10.1186/s12864-023-09758-8.

## Background

H3K9me3 and DNA methylation are two repressive marks that are associated with heterochromatin and transposable element (TE) repression [1, 2]. H3K9me3 and DNA methylation are positively correlated in fungi and plants [3, 4], but the relationship in mammals is more complicated [5]. The positive relationship between H3K9me3 and maintenance DNA methylation is particularly important at mouse developmental stages when the genome is broadly hypomethylated, including pre-implantation embryogenesis and primordial germ cell (PGC) development [6–8]. On the other hand, H3K9me3 is negatively associated with de novo DNA methylation during mouse spermatogenesis [9, 10]. Our recent study in mouse pre-implantation embryos discovered a highly positive correlation between H3K9me3 and DNA methylation at CpG-rich regions [6]. However, it is unclear whether the positive correlation between DNA methylation and H3K9me3 in CpG-rich regions exists for different biological processes, such as de novo DNA methylation processes during spermatogenesis.

In our previous study, we defined CpG-rich genomic loci with high H3K9me3 signals and DNA methylation levels as CHMs, which are widespread across the genome, not only in pericentromere-associated domains (PADs) but also in promoters and potential enhancers [6]. CHMs are important in the development of pre-implantation embryos, as they are hotspots for DNA methylation maintenance [6]. In addition, allele-specific CHMs include the majority of known imprinting control regions (ICRs) and dozens of ICR-like regions (ICRLRs), which play important roles in embryonic development by regulating the allele-specific expression of imprinted genes and transposable elements [6]. However, whether CHMs play regulatory roles in other biological processes during mouse development is still largely unknown. To address the above questions, we performed a comprehensive analysis to characterize the co-localization between H3K9me3 and DNA methylation at CpG-rich regions during multiple mouse developmental processes.

## Results

### CHMs are stable during mouse development

To explore the co-localization between H3K9me3 and DNA methylation, we collected public H3K9me3 chromatin immunoprecipitation sequencing (ChIP-seq) and whole-genome bisulfite sequencing (WGBS) data during mouse pre-implantation embryogenesis [11], PGC development [12], spermatogenesis [13, 14], retina development [15], heart and liver development after gastrulation [16–18] (Supplementary Table 1). By dividing the genome into three classes of regions according to the number of CpGs, we found that H3K9me3 signals and DNA methylation levels showed the highest positive correlations at CpG-rich regions in all processes (Supplementary Fig. S1A), which was consistent with our previous observation during pre-implantation embryogenesis [6]. This finding suggests that CHMs may play regulatory roles during multiple mouse development processes.

**Fig. 1:**
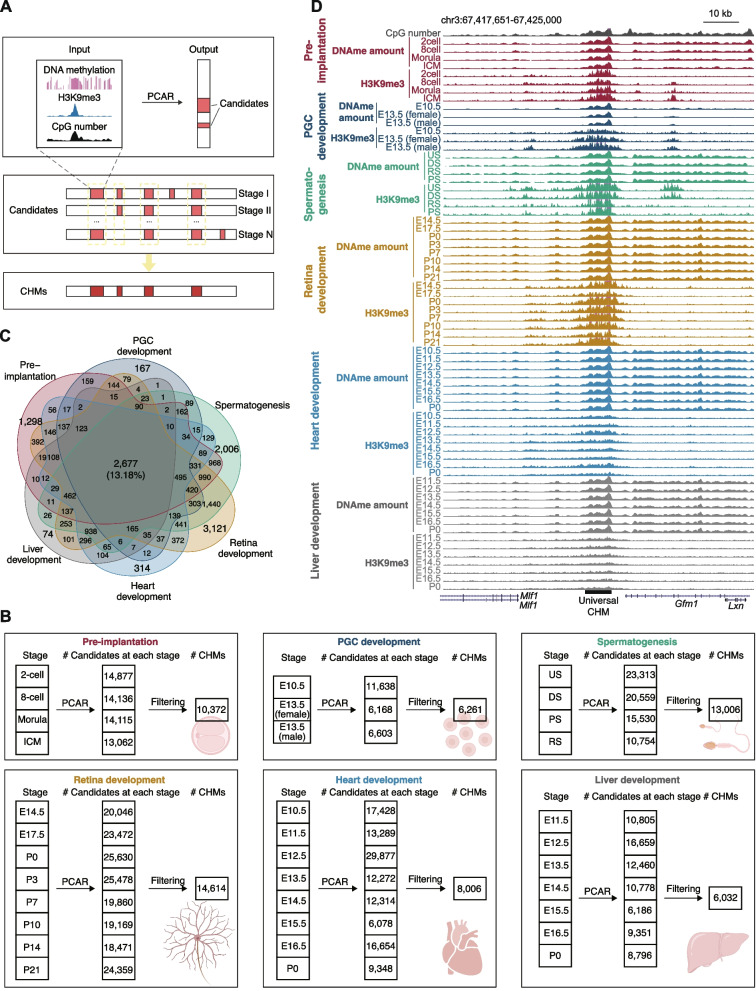
CHMs are stable during mouse development. **A** Definition of CHM candidates and CHMs. Candidates present in more than 2/3 stages of each developmental process were defined as CHMs. PCAR, a computational pipeline for CHM calling and scoring allele-specific regulatory potential. **B** Summary of candidates and CHMs in each developmental process. **C** Venn diagram showing the overlap of CHMs in 6 developmental processes. **D** The UCSC Genome Browser view of DNA methylation amount (DNAme amount), H3K9me3 signals, and CpG site number around a representative universal CHM. The scales of the CpG number track and DNAme amount tracks were 0–50, and those of the H3K9me3 tracks were 0–1. The genomic location of the universal CHM is indicated by a black rectangle. The DNA methylation amount represents the product of DNA methylation level and CpG number for each 1-kb window, and the H3K9me3 signal represents the ChIP-seq RPM

To investigate the features and potential roles of CHMs during mouse development, we identified CHMs in all developmental processes (Fig. 1A). For each developmental process, candidates were detected by a pipeline for CHM calling and scoring allele-specific regulatory potential, named PCAR [6] at each stage, and those present in more than two-thirds of the stages were defined as CHMs for this process. The number of identified CHMs ranged from 6,032 (liver development) to 14,614 (retina development) (Fig. 1B), suggesting that CHMs are widely spread in different developmental processes. In most cases, CHMs harbored significantly higher H3K9me3 signals and DNA methylation levels than non-ubiquitous candidates (Supplementary Fig. S1B and C), which were only present in less than two-thirds of stages of a developmental process.

To investigate whether H3K9me3 and DNA methylation always co-exist at CpG-rich regions, we further identified CH-nonMs (*i.e.*, CpG-rich regions marked by H3K9me3 but not DNA methylation) and CM-nonHs (*i.e.*, CpG-rich regions marked by DNA methylation but not H3K9me3) (see Methods for details). The total number of identified CM-nonHs (134,267) was larger than that of CHMs (20,313) and CH-non-Ms (14,707), indicating that both modifications do not always co-exist at CpG-rich regions. Nevertheless, 2,677 (13.18%) CHMs were present in all 6 developmental processes (termed universal CHMs), while only 41 (0.28%) and 1,206 (0.90%) CH-nonMs and CM-nonHs were found in all processes (Fig. 1C, D, Supplementary Fig. S1D and E). This finding indicates that CHM is one of the most stable modification patterns at CpG-rich regions during mouse development and that the identified universal CHMs are worth investigating for additional characterization of features and functions.

### Features of CHMs in distinct chromatin compartments

**Fig. 2:**
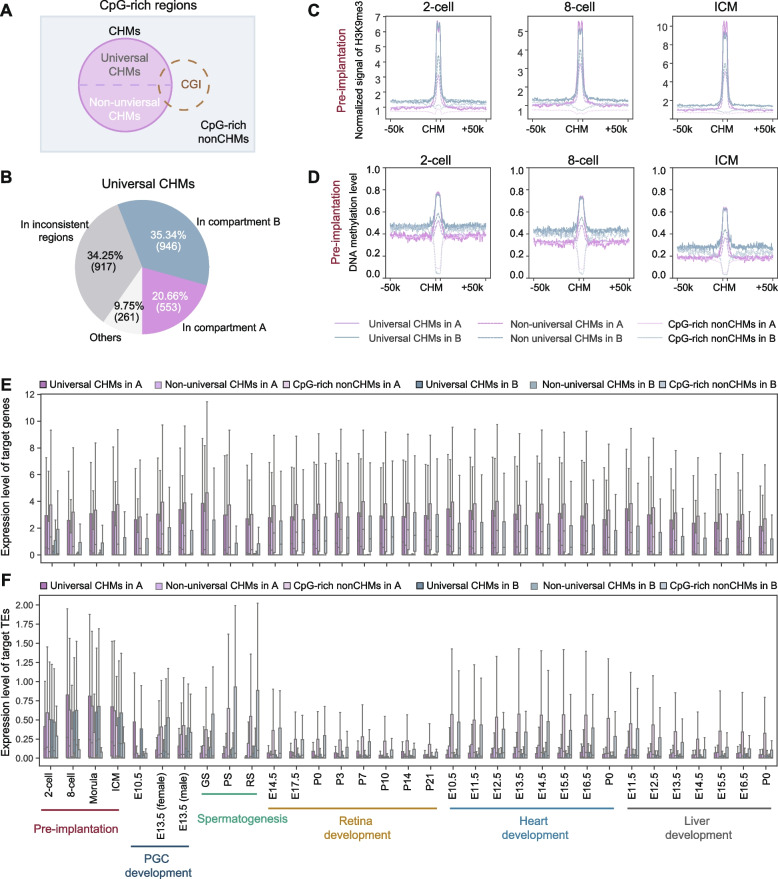
Environments of CHMs in compartment A and B. **A** Schematic representation of the relationship between universal CHMs, non-universal CHMs and CpG-rich nonCHMs. **B** Pie plot showing universal CHMs overlapping with compartment A and B. **C-D** Line plots showing H3K9me3 signals (**C**) and DNA methylation levels (**D**) surrounding universal CHMs (± 50 kb, solid), non-universal CHMs (± 50 kb, dashed) and CpG-rich nonCHMs (± 50 kb, dotted) in compartment A (pink) and B (blue) in pre-implantation embryogenesis. **E–F** Box plots showing the expression levels of potential target genes (**E**) and potential target TEs (**F**) of universal CHMs (dark), non-universal CHMs (mid-tone) and CpG-rich nonCHMs (light) in compartment A (pink) and B (blue). The expression level was calculated as the log_2_(TPM + 1). TPM, transcripts per kilobase million

CpG-rich regions play a crucial role in various aspects of gene regulation and genome functionality. One well-studied subset of these regions is the CpG islands (CGIs), which are generally unmethylated in healthy cells. In our study, we examined diverse types of CpG-rich regions, categorized based on CHM features. For clarity, we organized CpG-rich regions into two main groups: CHMs and non-CHM CpG-rich regions (see Methods for details). Additionally, we further segmented CHMs into universal CHMs and non-universal CHMs, defining the latter as a complementary set to universal CHMs across all six processes examined (Fig. 2A). To identify process-specific markers, we further categorized the non-universal CHMs into 1,298 pre-implantation-specific CHMs, 167 PGC development CHMs, 2,006 spermatogenesis-specific CHMs, 3,121 retina development-specific CHMs, 314 heart development-specific CHMs and 74 liver development-CHMs (see Methods for details). Given that H3K9me3 and DNA methylation are both repressive modification marks, we next investigated whether universal CHMs are exclusively located at heterochromatin. As compartment A and B derived from Hi-C datasets are regarded as euchromatin and heterochromatin respectively [19, 20], we calculated the percentages of universal CHMs in Hi-C compartments from 5 cell types (ESCs, NPCs, ncxNPCs, CNs, ncxCNs) [21] (see Methods for details). 946 (35.34%) universal CHMs were located in compartment B in all cell types, and 553 (20.66%) were located in compartment A in all cell types (Fig. 2B). In addition, 917 (34.25%) universal CHMs were located in different compartments in different cell types. The distributions of non-universal CHMs in compartment A and B were similar to those of universal CHMs (Supplementary Fig. S2A). Conversely, CpG-rich nonCHMs behaved differently, with 55.97% (12,351) located in compartment A, while only a mere 2.90% (639) were located in compartment B (Supplementary Fig. S2B). Our results demonstrated that universal CHMs and non-universal CHMs were not exclusively located in heterochromatin, while CpG-rich nonCHMs prefer euchromatin. Upon further analysis, we found that retina development-specific CHMs tend to be more prevalent in the inconsistent compartment A in neural cells, especially in NPCs and CNs, comparing to other types of CHMs (Supplementary Fig. S2C). This observation subtly hints at the potential functional significance of process-specific CHMs.

To characterize the features of universal CHMs, non-universal CHMs and CpG-rich nonCHMs in compartment A and B, we displayed the H3K9me3 signals and DNA methylation levels around universal CHMs in two compartments separately. As expected, we observed much higher H3K9me3 signals at CHMs than adjacent regions in both compartments during all developmental processes, while the H3K9me3 signals at adjacent regions in compartment B were relatively higher than those in compartment A (Fig. 2C and Supplementary Fig. S2D). Furthermore, in most instances, the H3K9me3 signals of universal CHMs were slightly elevated compared to those of non-universal CHMs. However, the patterns of DNA methylation levels around CHMs were complicated (Fig. 2D and Supplementary Fig. S2E). CHMs displayed much higher DNA methylation levels than adjacent regions during pre-implantation embryogenesis and PGC development, while the DNA methylation level differences between CHMs and adjacent regions were weak for other developmental processes, mainly due to the high background DNA methylation level across the mammalian genome in most tissues [1]. Strikingly, DNA methylation levels at adjacent regions of universal CHMs and non-unversal CHMs were even lower in compartment B than in compartment A at some stages of spermatogenesis, retina, heart, and liver development. Furthermore, as expected, the H3K9me3 signals and DNA methylation levels in CpG-rich nonCHM regions were much lower compared to those in the other two types of CpG-rich regions. Taken together, the findings indicate that the key feature of CHMs as genomic islands is that H3K9me3 signals are much higher than those in adjacent regions, regardless of compartment A or B.

To investigate whether these CpG-rich regions play regulatory roles, we first examined their distances to genes and TEs in compartment A and B respectively. Compared with CpG-rich nonCHMs, CHMs displayed much larger distances to their nearest gene transcription start sites (TSSs), especially for those in compartment B (Supplementary Fig. S3A). For the distances to the nearest TEs, there were no clear differences between CHMs and CpG-rich nonCHMs in both compartment A and B (Supplementary Fig. S3A; see Methods for details). Then we examined the expression levels of potential target genes and TEs of CHMs and CpG-rich nonCHMs (see Methods for details). No matter in compartment A or B, the expression levels of CHMs’ potential target genes and TEs were significantly lower than those of CpG-rich nonCHMs’ potential targets (Fig. 2E, F), indicating that CHMs play a repressive regulatory role, consistent with the known properties of H3K9me3. The functional enrichment analysis showed that the potential target genes of universal CHM in compartment A were enriched in the regulation of developmental process, while non-universal CHMs in compartment A were enriched in metabolic processes, whereas universal CHMs and non-universal CHMs in compartment B were enriched in sensory perception of stimuli (Supplementary Fig. S3B, Supplementary Table 2). The target genes of CpG-rich nonCHM regions were enriched in cellular metabolic processes in compartment A and associated with nervous system development in compartment B. We also observed that pre-implantation-specific CHMs in compartment A have target genes enriched in cellular metabolic processes, while spermatogenesis-specific CHMs in the same compartment are enriched in female gamete generation (Supplementary Fig. S3C, Supplementary Table 3). This points to a potential inhibitory role of process-specific CHMs in compartment A.

### Enrichment of CHMs in IAP subfamilies

**Fig. 3:**
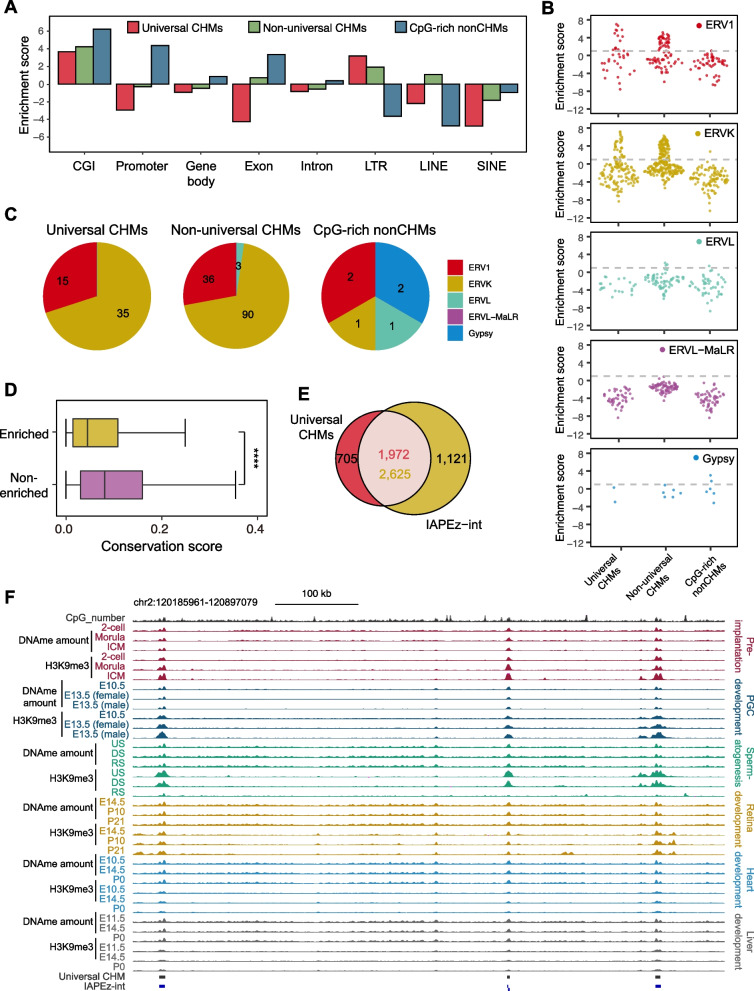
Universal CHMs tend to be enriched in IAP subfamilies. **A** Bar plot showing the enrichment of universal CHMs (red), non-universal CHMs (green) and CpG-rich nonCHMs (blue) in CGI, promoter, gene body, exon, intron, LTR, LINE and SINE regions. The enrichment score represents the log_2_-transformed observed overlapping length/the expected overlapping length ratio. **B** Sina plots showing the enrichment scores of universal CHMs, non-universal CHMs and CpG-rich nonCHMs in LTR subfamilies. Element parts of each LTR subfamily, for example, IAPEz-int, IAPLTR1_Mm and IAPLTR1a_Mm, were represented by a dot respectively. Element parts from different LTR subfamilies were differentiated using distinct colors. **C** Pie plot showing the components of enriched element parts of LTR subfamilies in universal CHMs (left), non-universal CHMs (middle) and CpG-rich nonCHMs (right). Element parts of the LTR subfamily with an enrichment score ≥ 1 was considered to be enriched, which were dots above the dashed line in Fig. 3B. **D** Boxplot showing the conservation score of element parts of LTR subfamilies enriched or non-enriched in universal CHMs and non-universal CHMs. **E** Venn diagram showing the overlap between universal CHMs (red) and IAPEz-int (yellow). **F** The UCSC Genome Browser view of DNAme amount, H3K9me3 signals and CpG site number around representative universal CHMs and IAPEz-int. The scales of the CpG number track and DNA methylation amount tracks were 0–50, and those of the H3K9me3 tracks were 0–1. The genomic locations of the universal CHMs and IAPEz-int are indicated by black and blue rectangles, respectively. The DNA methylation amount represents the product of DNA methylation level and CpG number for each 1-kb window, and the H3K9me3 signal represents the ChIP-seq RPM

To further depict the genomic distribution of universal CHMs, we investigated their regional enrichment. As expected, universal CHMs, non-universal CHMs, and CpG-rich nonCHMs were enriched in CpG islands (CGIs) (Fig. 3A). Universal CHMs and non-universal CHMs were enriched in long terminal repeats (LTRs), non-universal CHMs were enriched in Long Interspersed Nuclear Element (LINE), while CpG-rich nonCHMs were enriched in promoters and exons (Fig. 3A). In addition, on most occasions, the regional enrichments of universal CHMs and non-universal CHMs in compartment A displayed similarities, as did non-universal CHMs and CpG-rich nonCHMs in compartment B (Supplementary Fig. S4A). The process-specific CHMs exhibited diverse patterns of enrichment in certain genomic regions, including the enrichment in promoters and exons of retina-specific and liver-specific CHMs, while the enrichment in LINE of pre-implantation-specific and spermatogenesis-specific CHMs (Supplementary Fig. S4B). We then investigated the enrichment of CHMs in families and subfamilies of repetitive elements and found that universal CHMs and non-universal CHMs were highly enriched in subfamilies of two LTR families, ERVK and ERV1, compared to CpG-rich nonCHMs (Fig. 3B, C). In contrast, universal CHMs were not enriched in two other types of retrotransposons, LINEs and SINEs (Fig. 3A, Supplementary Fig. S4C and D). Non-universal CHMs exhibited a slight enrichment in LINEs compared to the other two types of CpG-rich regions (Supplementary Fig. S4C). Interestingly, compared with LTR subfamilies not enriched in universal CHMs and non-universal CHMs, element parts of LTR subfamilies they enriched in were less well conserved in evolution (Fig. 3D; see Methods for details), indicating that CHMs may be involved in the silencing of evolutionarily younger LTRs.

We next investigated the detailed relationship between CHMs and enriched LTR subfamilies. By displaying the overlapping ratios and enrichment scores, we found a significant overlap between universal CHMs and a specific subgroup of IAP ERVs (Intracisternal A Particle Endogenous Retroviruses), namely the IAPEz-int (and its cognate LTRs IAPLTR1a-Mm and IAPLTR1_Mm), with an enrichment score above 1 and an overlap ratio exceeding 10% (Fig. 3E, F and Supplementary Fig. S4E). However, for non-universal CHMs, no subfamilies of either LTR or LINE exhibited significant enrichment overlap (Supplementary Fig. S4F, SG). For the IAPEz-int, as most of the members overlapped with universal CHMs (Fig. 3E), we suspected that the remaining members of IAPEz-int overlapped with CHM candidates identified in at least one stage. The percentages of the IAPEz-int overlapping with or having at least one CHM candidate nearby (± 2 kb) were indeed 98.93% when considering all CHM candidates, demonstrating the role of CHMs in silencing specific ERVK subfamilies globally. It was previously reported that a significant overlap existed between DMR and the IAPEz-int sub-family, which was associated with Dnmt1’s de novo activity during the early stages of development [22]. Our findings suggested that while there were CHMs exhibiting inconsistency, such as H3K9me3 or 5mC gain or loss particularly during pre-implantation and PGC development, the predominant overlap of IAPEz-int and CpG-rich regions was observed at CHM consistent regions (Supplementary Fig. S4H-J). This implied that IAPEz-int required both H3K9me3 and 5mC for their repression during development.

### DNA sequence features of CHMs

**Fig. 4:**
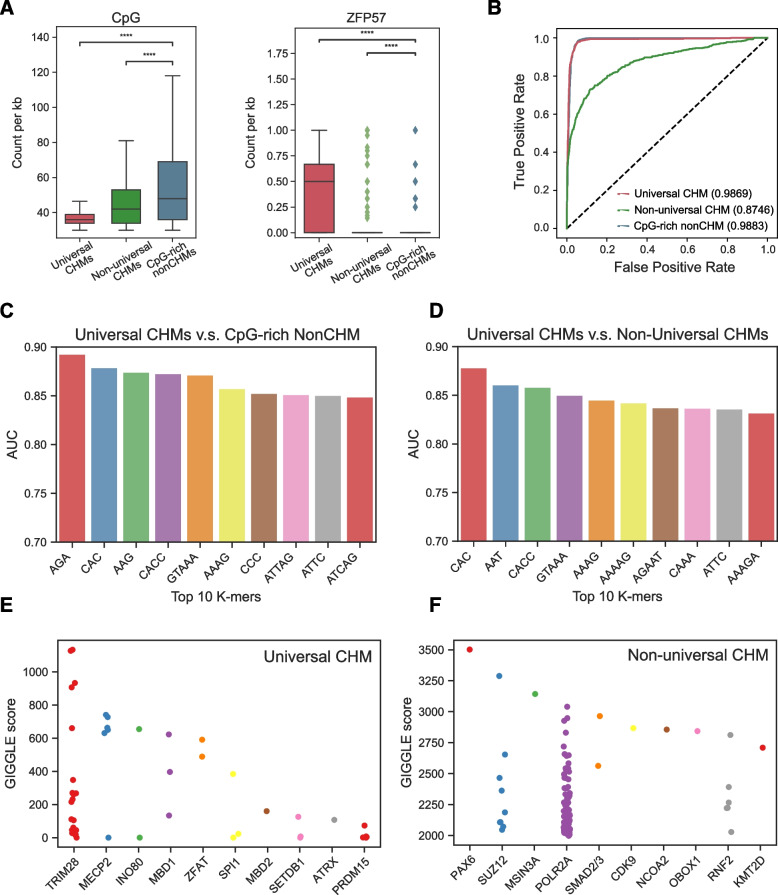
Potential formation mechanisms of universal CHMs. **A** Box plots showing the frequency of CpGs (left) and methylated ZFP57 motifs (right) in universal CHM CpG-rich 1-kb bins and CpG-rich nonCHMs. CpG sites in ZFP57 motifs were considered to be methylated if the DNA methylation level ≥ 0.5 in more than 2/3 stages. The frequencies of 1-kb bins from the same universal CHMs were averaged to one number. **B** ROC curve showing the prediction of universal CHMs (red), non-universal CHMs (green), CpG-rich nonCHMs (blue) using Nucleotide Transformer fine-tuned with a MLP model. The Area Under the Curve (AUC) scores were indicated in the brackets. **C-D** Bar plot showing the AUC of top 10 k-mers with the best performance in distinguishing universal CHMs from CpG-rich nonCHMs (**C**), and universal CHMs from non-universal CHMs (**D**). **E–F** Strip plot showing the top 10 transcription factors most similar to the CpG-rich 1 kb-bins of universal CHMs (**E**) and non-universal CHMs (**F**). CpG-rich 1 kb-bins were defined as genomic 1 kb-bins with more than 30 CpGs inside

To explore whether universal CHMs have distinct DNA sequence features, we compared the nucleotide composition between universal CHMs, non-universal CHMs and CpG-rich nonCHMs. We found that universal CHMs had the lowest CpG frequencies (Fig. 4A), demonstrating that universal CHMs are a subset of CpG-rich regions with lower CpG frequency. Given that ZFP57 is an ICR-related regulator whose DNA binding motif is TGCmCGC [23, 24], we examined its abundance in all types of CpG-rich regions, and found a significantly higher count of ZFP57 motif hits in universal CHMs than in other two CpG-rich regions (Fig. 4A). Our results indicate that universal CHMs indeed differ from other two CpG-rich regions in sequence features.

To detect whether universal CHMs, non-universal CHMs and CpG-rich nonCHMs have distinct features of DNA sequences, we fine-tuned Nucleotide Transformer [25] with a multi-layer perceptron (MLP) (see Methods for details). Optimal accuracy on the test data was achieved with the following parameters: 100 epochs, a batch size of 50, a learning rate of 0.001, and the usage of the 20th embed layer of the Nucleotide Transformer. Upon evaluating the performance of the fine-tuned model, we conducted a receiver operating characteristic (ROC) analysis for each of the three CpG-rich region types, comparing each against the other two. Intriguingly, the area under the curve (AUC) amounted to 0.9869 for universal CHMs, 0.8746 for non-universal CHMs, and 0.9883 for CpG-rich nonCHMs (Fig. 4B). In terms of precision-recall analysis, the area under the precision-recall curve (AUPRC) yielded values of 0.9085 for universal CHMs, 0.6061 for non-universal CHMs, and 0.9954 for CpG-rich nonCHMs. These results suggested that each type of CpG-rich region has specific DNA sequence features.

To investigate the exact DNA features of universal CHMs, we next generated all possible DNA k-mers (1 ≤ k ≤ 6) and examined their occurrences between universal CHMs and CpG-rich nonCHMs. We conducted a receiver operating characteristic (ROC) analysis to pinpoint k-mers whose frequency of occurrence could differentiate between universal CHMs and CpG-rich nonCHMs, universal CHMs and non-universal CHMs, as well as non-universal CHMs and CpG-rich nonCHMs (see Methods for details). The top 10 k-mers adeptly distinguished universal CHMs from the other two types of CpG-rich regions well, as evidenced by AUC values ranging between 0.85 and 0.90 (Fig. 4C, 4D). When attepting to distinguish between non-universal CHMs and CpG-rich nonCHMs, AUC scores were slightly lower, ranging from 0.70 to 0.80 (Supplementary Fig. S5A). Among the top 10 k-mers that best differentiated universal CHMs from CpG-rich nonCHMs best, 7 were more frequently found in the universal CHMs, while the remaining 3 were more common in CpG-rich nonCHMs (Supplementary Fig. S5B). There was also a significant difference in the frequency of the top 10 k-mers that distinguished universal CHMs from non-universal CHMs, as well as non-universal CHMs from CpG-rich nonCHMs, across these CpG-rich regions (Supplementary Fig. S5C, D). This revealed a distinct difference in DNA sequence features among the three types of CpG-rich regions. Such a sequence preference in universal CHMs was also discernable in IAPEz, which extensively overlapped with universal CHMs (Supplementary Fig. S5E), suggesting that the sequence preference might be derived from this ERVK subfamily. These results indicating the roles of DNA sequence preferences in CHM determination.

To investigate the differences in transcription factor (TF) binding preferences between different types of CpG-rich regions, we applied the Cistrome toolkit [26, 27] to identify potential binding TFs or chromatin regulator (CR). We found that universal CHMs had a higher potential to be bound by TRIM28, MECP2, INO80, MBD1, etc. (Fig. 4E, Supplementary Table 4), which included known methyltransferases of H3K9me3, DNA methylation binding factors, suggesting their roles in maintaining the co-localization of two epigenetic modifications. However, non-universal CHMs exhibited a heightened potential for interaction with transcriptionally active factors such as POLR2A (Fig. 4F). CpG-rich nonCHMs, on the other hand, had a higher potential to be bound by general transcription factors, such as MED1 and GTF2B (Supplementary Fig. S5F). Process-specific CHMs tended to be associated with corresponding transcription factors. For instance, GATA4, which is related to myocardial differentiation and function, was likely to bind to heart development-specific CHMs. Similarly, FOXA, instrumental in liver differentiation, was found to associate with liver development-specific CHMs (Supplementary Fig. S5H, Supplementary Table 4). Our results indicate that there are distinct transcription factor binding potentials between different types of CpG-rich regions, especially universal CHMs and nonCHM CpG-rich regions.

## Discussion

H3K9me3 and DNA methylation frequently co-localize to silence genes and transposable elements in heterochromatin, which is usually GC-poor [28]. However, our previous study in mouse embryos revealed their co-localization at CpG-rich regions, termed CHMs, and suggested that those regions can escape DNA demethylation during pre-implantation embryogenesis [6]. Certain types of CpG-rich regions, such as CpG islands (CGIs), which are typically conserved and associated with gene promoters, have been extensively studied. These regions are often free from DNA methylation. In this study, we confirmed the co-localization of H3K9me3 and DNA methylation in CpG-rich regions in multiple mouse developmental processes and termed the consistently co-localized regions as universal CHMs. Universal CHMs were distinct from other CpG-rich regions, *i.e.*, CpG-rich nonCHMs, in three aspects. First, many more universal CHMs in proportion were located consistently in compartment B than CpG-rich nonCHMs (35.34% *vs*. 2.90%). Second, universal CHMs were specifically enriched in LTRs, while CpG-rich nonCHMs were specifically enriched in gene promoters and exons. Third, the DNA sequence features of those two types of CpG-rich regions differed significantly. This study broadens our understanding of the regulatory roles of CpG-rich regions.

Some types of repetitive elements are known to be suppressed through the co-occupancy of H3K9me3 and DNA methylation [29]. Our study highlighted the co-localization of H3K9me3 and DNA methylation in the ERVK and ERV1 families, particularly in the ERVK subfamily IAPEz-int. Those evolutionarily younger repeat subfamilies are thought to be regulated by KRAB-containing zinc finger proteins (KZFPs) and their cofactor TRIM28 [30, 31], whose binding sites preferentially overlap with universal CHMs, suggesting a similar regulatory mechanism for universal CHMs.

## Conclusions

Our research provides a comprehensive analysis of H3K9me3 and DNA methylation co-marked CpG-rich regions (CHMs) across six distinct mouse developmental processes. We have discovered that the co-occurrence of H3K9me3 and DNA methylation represents one of the most consistent modification patterns within CpG-rich regions, with more than 2,600 CHMs observed throughout all examined developmental stages. Notably, CHMs are characterized by significantly higher H3K9me3 signals when compared to surrounding regions, with a large portion located in euchromatin. Our findings also suggest a potential role of sequence preferences in determining CHM characteristics and hint at the possible involvement of CHMs in silencing evolutionarily younger LTRs, particularly within certain ERVK subfamilies. This study elucidates the distinct features of CHMs during multiple developmental processes and enriches our understanding of the regulatory roles of CpG-rich regions in mammalian development. Further research may lead to new insights into the intricate epigenetic regulation of developmental processes and contribute to our knowledge of the biological significance of these modifications.

## Methods

### Data processing and normalization for ChIP-seq, RNA-seq, and WGBS

ChIP-seq reads were aligned to the mouse genome mm10 using Bowtie2 (v2.4.2) [32] with default parameters. Signal tracks for each sample were generated using the MACS2 (v2.1.1.20160309) [33] pileup function and were saved as reads per million (RPM). ChIP-seq biological replicates were pooled. RNA-seq reads were aligned to the mouse genome mm10 using HISAT2 (v2.1.0) [34] with default parameters. The expression levels for all RefSeq genes were quantified as TPM values using StringTie (v2.1.4) [35], and the TPM values of replicates were averaged. All of the WGBS reads were first processed using Trim galore (v0.6.6) to trim low-quality reads. The trimmed WGBS reads were then mapped to the mouse genome mm10 using bsmap, and the methylation level of each CpG site was estimated using mcall. Both bsmap and mcall were from MOABS (v1.3.2) [36].

### Calculation of DNA methylation amount (DNAme amount)

We generated genome-wide sliding windows with 1 kb as the size and 10 bp as the step and calculated the DNA methylation level (methylated CpG/total CpG) and CpG number for each 1-kb window separately. Then we multiplied the DNA methylation level and CpG number for each 1-kb window as the DNA methylation amount (DNAme amount) for the center position of the given window.

### CHM-related terminology

PCAR (v0.1.0) [6] was used to call candidates with default parameters. For each developmental process, candidates existing in more than 2/3 stages were defined as CHMs, while others were defined as non-ubiquitous candidates. Neighboring CHMs within 2 kb were merged. CHMs existing in all 6 processes were defined as universal CHMs. Non-universal CHMs were defined as the complementary set to universal CHMs across all six processes. CH-nonMs and CM-nonHs were called using PCAR with the parameters “-D CHnonM” or “-D CMnonH” respectively. 1-kb bins with more than 30 CpGs were selected as CpG-rich regions. CpG-rich regions without CHM candidates within 2 kb were defined as CpG-rich nonCHMs. CHMs existing in only one certain process were defined as process-specific CHMs. For example, CHMs only exited during heart development were defined as heart development-specific CHMs.

### Compartment calling

The annotations of compartment A and B in mouse ESCs, in vitro differentiated neural progenitor cells (NPCs), in vitro differentiated cortical neurons (CNs), NPCs purified in vivo from the E14.5 neocortex (ncxNPCs), and CNs purified in vivo from the E14.5 neocortex (ncxCNs) were downloaded from Bonev et al*.* (2017) [21]. The A or B compartments from 5 tissues were intersected, respectively. Regions that were not either A or B in the 5 cell types were intersected and classified into the “other” group. Regions that were inconsistent in the 5 cell types were classified into the “inconsistent” group. Inconsistent compartment A and B regions were defined as genome regions which were not consistent to be compartment A or compartment B across the 5 cell types (ESCs, NPCs, CNs, ncxNPCs and ncxCNs).

### Assembly of TE transcripts

Transposable element (TE) transcripts during mouse development were constructed as in ref. [37]. The RNA-seq reads in each developmental stage were mapped to the mm10 genome using STAR (v2.7.4a) [38] with the following parameters: “–outSAMtype BAM SortedByCoordinate –outFilterMultimapNmax 500 –outSAMattributes NH HI NM MD XS AS –readFilesCommand zcat”. Transcript assembly of each RNA-seq sample was performed using StringTie (v2.1.4) [35] with the following parameters: “-j 2 -s 5 -f 0.05 -c 2”. Assembled transcripts from multiple RNA-seq samples were merged using TACO (v0.7.3) [39] with default parameters. Transcripts whose exons overlapped with transposable elements but not the exons of RefSeq protein-coding genes were regarded as TE transcripts.

### Calculation conservation score for TEs

The conservation scores for Transposable Elements (TEs) were obtained from the UCSC Table Browser (https://genome.ucsc.edu/cgi-bin/hgTables) [40]. The scores were derived from the mm10 assembly, specifically from the “Comparative Genomics” group, the “Conservation” track, and the “60 Vert. Cons (phastCons60way)” table. These scores encompassed three subsets: Glires, Euarchontoglires, and placental mammal.

### Potential target genes/TEs

We defined genes whose TSSs were located within 10 kb of universal CHMs, non-universal CHMs, CpG-rich non-CHMs or process-specific CHMs as their potential target genes and TEs overlapping with universal CHMs, non-universal CHMs and CpG-rich nonCHMs as their potential target TEs.

### Genomic enrichment analysis

The enrichment of universal CHMs, non-universal CHMs, CpG-rich nonCHMs and process-specific CHMs in genomic features such as CGI, promoter, gene body, exon, intron, LTR, LINE and SINE was calculated. We used the log_2_-transformed “observed overlapping length ratio” divided by the “expected overlapping length ratio” as the enrichment score. The observed overlapping ratio was the proportion of the length of universal CHMs, non-universal CHMs, CpG-rich nonCHMs or process-specific CHMs overlapping with features to the total length of universal CHMs, non-universal CHMs, CpG-rich nonCHMs or process-specific CHMs. The expected overlapping length ratio was the proportion of each feature’s total length, for example, total length of promoters, to the whole mm10 genome.

### Functional annotation

Functional annotation clustering analysis was performed using the Database for Annotation, Visualization and Integrated Discovery (DAVID) Bioinformatics Resource 6.8 [41]. For each functional cluster from “GOTERM_BP_ALL”, one representative Gene Ontology (GO) term was selected, and its -log10(*p*-value) was plotted to indicate the significance. Regarding the CpG-rich nonCHMs, the number of their target genes in compartment A exceeded the upper limit of 3,000 as set by DAVID. We therefore randomly selected 3,000 genes from the target genes of CpG-rich nonCHM regions five times. These selections were individually processed through DAVID each time and the resulting GO terms were combined. Due to the limited number of CHMs of each process-specific CHM classes, we could only obtain significant GO terms enriched in pre-implantation-specific CHMs and PGC development CHMs.

### Fine-tuning the nucleotide transformer

We utilized the pre-trained '2B5_multi_species' Nucleotide Transformer model [25], setting the 'max_positions' parameter to 171. This was calculated based on the 1-kb sequence length we used and the 6-mer maximum k-mer length utilized by the Nucleotide Transformer. For our predictions, we used 3,402 CpG-rich 1 kb-bins of universal CHMs, 2,392 CpG-rich 1 kb-bins of non-universal CHMs, and 22,069 CpG-rich nonCHMs, excluding 7 CpG-rich nonCHMs containing N sites. We stored the embedding results from layers 10 to 20 during the prediction process for separate fine-tuning. 20% of the labeled embedding scores were randomly selected as a test dataset, with the remaining data split into a 4:1 ratio for training and validation datasets. A Multi-Layer Perceptron (MLP) model was utilized to fine-tune the embedding scores across all combinations of hyperparameters: embedding layers of 10, 15, 20; batch sizes of 50, 100, 500; epoch numbers of 10, 50, 100; and learning rates of 0.001, 0.0001, 0.00001. The MLP model was constructed with a flatten layer, a linear layer (with 2,560 inputs and 512 outputs), a ReLU layer, and a final linear layer (with 512 inputs and 3 outputs for class probability determination). After extensive fine-tuning, the optimal parameters for the highest test dataset accuracy (0.9616) were found to be a learning rate of 0.001, 100 epochs, a batch size of 50, and using the 20^th^ embedding layer.

### ROC analysis of k-mers

The frequencies of each possible k-mer (1 ≤ k ≤ 6) were calculated for universal CHM, non-universal CHMs and CpG-rich nonCHM sequences. For each possible k-mer, we labeled universal CHM sequences as “1” and CpG-rich non-CHM sequences as “0” and used the sequence labels and k-mer frequency to calculate the AUC score with Scikit-learn (sklearn, v1.1.3) [42]. The frequency and AUC score of each k-mer were equivalent to those of its reverse complementary k-mer. This process was similarly carried out for universal CHMs versus non-universal CHMs, as well as non-universal CHMs versus CpG-rich nonCHMs.

### Statistical analysis

Statistical analysis was performed using R or SciPy (v1.5.4) [43], and the statistical details are shown in the figure legends. Asterisks represent statistical significance (****: *p-value* < 0.0001; ***: *p-value* < 0.001; **: *p-value* < 0.01; *: *p-value* < 0.05; n.s.: not significant).

### Supplementary Information

**Additional file 1:** **Supplementary Figure S1.** CHMs are one of the most stable forms at CpG-rich regions during mouse development. **Supplementary Figure S2.** Environments of CHMs in compartment A and B. **Supplementary Figure S3.** Potential functions of CHMs in compartment A and B. **Supplementary Figure S4.** Enrichment of universal CHMs in repeats. **Supplementary Figure S5.** Potential formation mechanisms of universal CHMs.

**Additional file 2:** **Supplementary Table S1.** Public high through-put sequencing data used in this study. **Supplementary Table S2. **Biological Process (BP) GO term enrichment analysis of potential target genes of universal CHMs, non-universal CHMs and CpG-rich nonCHMs in compartment A and B. **Supplementary Table S3.** Biological Process (BP) GO term enrichment analysis of potential target genes of pre-implantation-specific CHMs and spermatogenesis-specific CHMs in compartment A and B. **Supplementary Table S4.** Results of Toolkit for Cistrome Data Browser to find significant factors binding overlap with universal CHMs, non-universal CHMs, CpG-rich nonCHMs and process-specific CHMs.

## Data Availability

All scripts used for the analysis described are available on GitHub (https://github.com/TongjiZhanglab/CHMs_during_mouse_development). The datasets supporting the conclusions of this article are included within the article and its additional files.

## References

[1] Greenberg MVC, Bourc'his D. The diverse roles of DNA methylation in mammalian development and disease. Nat Rev Mol Cell Biol. 2019;20(10):590–607. 10.1038/s41580-019-0159-6 31399642

[2] Jambhekar A, Dhall A, Shi Y (2019). Roles and regulation of histone methylation in animal development. Nat Rev Mol Cell Biol.

[3] Jackson JP, Lindroth AM, Cao X, Jacobsen SE (2002). Control of CpNpG DNA methylation by the KRYPTONITE histone H3 methyltransferase. Nature.

[4] Tamaru H, Selker EU (2001). A histone H3 methyltransferase controls DNA methylation in Neurospora crassa. Nature.

[5] Janssen SM, Lorincz MC (2022). Interplay between chromatin marks in development and disease. Nat Rev Genet.

[6] Yang H, Bai D, Li Y, Yu Z, Wang C, Sheng Y, Liu W, Gao S, Zhang Y (2022). Allele-specific H3K9me3 and DNA methylation co-marked CpG-rich regions serve as potential imprinting control regions in pre-implantation embryo. Nat Cell Biol.

[7] Leung D, Du T, Wagner U, Xie W, Lee AY, Goyal P, Li Y, Szulwach KE, Jin P, Lorincz MC (2014). Regulation of DNA methylation turnover at LTR retrotransposons and imprinted loci by the histone methyltransferase Setdb1. Proc Natl Acad Sci U S A.

[8] Liu S, Brind'Amour J, Karimi MM, Shirane K, Bogutz A, Lefebvre L, Sasaki H, Shinkai Y, Lorincz MC (2014). Setdb1 is required for germline development and silencing of H3K9me3-marked endogenous retroviruses in primordial germ cells. Genes Dev.

[9] Yamanaka S, Nishihara H, Toh H, Eijy Nagai LA, Hashimoto K, Park SJ, Shibuya A, Suzuki AM, Tanaka Y, Nakai K (2019). Broad Heterochromatic Domains Open in Gonocyte Development Prior to De Novo DNA Methylation. Dev Cell.

[10] Shirane K, Miura F, Ito T, Lorincz MC (2020). NSD1-deposited H3K36me2 directs de novo methylation in the mouse male germline and counteracts Polycomb-associated silencing. Nat Genet.

[11] Wang C, Liu X, Gao Y, Yang L, Li C, Liu W, Chen C, Kou X, Zhao Y, Chen J (2018). Reprogramming of H3K9me3-dependent heterochromatin during mammalian embryo development. Nat Cell Biol.

[12] Hill PWS, Leitch HG, Requena CE, Sun Z, Amouroux R, Roman-Trufero M, Borkowska M, Terragni J, Vaisvila R, Linnett S (2018). Epigenetic reprogramming enables the transition from primordial germ cell to gonocyte. Nature.

[13] Hasegawa K, Sin HS, Maezawa S, Broering TJ, Kartashov AV, Alavattam KG, Ichijima Y, Zhang F, Bacon WC, Greis KD (2015). SCML2 establishes the male germline epigenome through regulation of histone H2A ubiquitination. Dev Cell.

[14] Liu Y, Zhang Y, Yin J, Gao Y, Li Y, Bai D, He W, Li X, Zhang P, Li R (2019). Distinct H3K9me3 and DNA methylation modifications during mouse spermatogenesis. J Biol Chem.

[15] Aldiri I, Xu B, Wang L, Chen X, Hiler D, Griffiths L, Valentine M, Shirinifard A, Thiagarajan S, Sablauer A (2017). The Dynamic Epigenetic Landscape of the Retina During Development, Reprogramming, and Tumorigenesis. Neuron.

[16] Consortium EP (2012). An integrated encyclopedia of DNA elements in the human genome. Nature.

[17] Sloan CA, Chan ET, Davidson JM, Malladi VS, Strattan JS, Hitz BC, Gabdank I, Narayanan AK, Ho M, Lee BT (2016). ENCODE data at the ENCODE portal. Nucleic Acids Res.

[18] Davis CA, Hitz BC, Sloan CA, Chan ET, Davidson JM, Gabdank I, Hilton JA, Jain K, Baymuradov UK, Narayanan AK (2018). The Encyclopedia of DNA elements (ENCODE): data portal update. Nucleic Acids Res.

[19] Bonev B, Cavalli G (2016). Organization and function of the 3D genome. Nat Rev Genet.

[20] Falk M, Feodorova Y, Naumova N, Imakaev M, Lajoie BR, Leonhardt H, Joffe B, Dekker J, Fudenberg G, Solovei I (2019). Heterochromatin drives compartmentalization of inverted and conventional nuclei. Nature.

[21] Bonev B, Mendelson Cohen N, Szabo Q, Fritsch L, Papadopoulos GL, Lubling Y, Xu X, Lv X, Hugnot JP, Tanay A (2017). Multiscale 3D Genome Rewiring during Mouse Neural Development. Cell.

[22] Haggerty C, Kretzmer H, Riemenschneider C, Kumar AS, Mattei AL, Bailly N, Gottfreund J, Giesselmann P, Weigert R, Brandl B (2021). Dnmt1 has de novo activity targeted to transposable elements. Nat Struct Mol Biol.

[23] Quenneville S, Verde G, Corsinotti A, Kapopoulou A, Jakobsson J, Offner S, Baglivo I, Pedone PV, Grimaldi G, Riccio A (2011). In embryonic stem cells, ZFP57/KAP1 recognize a methylated hexanucleotide to affect chromatin and DNA methylation of imprinting control regions. Mol Cell.

[24] Liu Y, Toh H, Sasaki H, Zhang X, Cheng X (2012). An atomic model of Zfp57 recognition of CpG methylation within a specific DNA sequence. Genes Dev.

[25] Dalla-Torre H, Gonzalez L, Revilla JM, Carranza NL, Grzywaczewski AH, Oteri F, Dallago C, Trop E, Sirelkhatim H, Richard G, et al. The Nucleotide Transformer: Building and Evaluating Robust Foundation Models for Human Genomics. bioRxiv. 2023:2023.2001.2011.523679.

[26] Mei S, Qin Q, Wu Q, Sun H, Zheng R, Zang C, Zhu M, Wu J, Shi X, Taing L (2017). Cistrome Data Browser: a data portal for ChIP-Seq and chromatin accessibility data in human and mouse. Nucleic Acids Res.

[27] Zheng R, Wan C, Mei S, Qin Q, Wu Q, Sun H, Chen CH, Brown M, Zhang X, Meyer CA (2019). Cistrome Data Browser: expanded datasets and new tools for gene regulatory analysis. Nucleic Acids Res.

[28] Jabbari K, Chakraborty M, Wiehe T (2019). DNA sequence-dependent chromatin architecture and nuclear hubs formation. Sci Rep.

[29] Karimi MM, Goyal P, Maksakova IA, Bilenky M, Leung D, Tang JX, Shinkai Y, Mager DL, Jones S, Hirst M (2011). DNA methylation and SETDB1/H3K9me3 regulate predominantly distinct sets of genes, retroelements, and chimeric transcripts in mESCs. Cell Stem Cell.

[30] Rowe HM, Jakobsson J, Mesnard D, Rougemont J, Reynard S, Aktas T, Maillard PV, Layard-Liesching H, Verp S, Marquis J (2010). KAP1 controls endogenous retroviruses in embryonic stem cells. Nature.

[31] Coluccio A, Ecco G, Duc J, Offner S, Turelli P, Trono D (2018). Individual retrotransposon integrants are differentially controlled by KZFP/KAP1-dependent histone methylation, DNA methylation and TET-mediated hydroxymethylation in naive embryonic stem cells. Epigenetics Chromatin.

[32] Langdon WB (2015). Performance of genetic programming optimised Bowtie2 on genome comparison and analytic testing (GCAT) benchmarks. BioData Min.

[33] Zhang Y, Liu T, Meyer CA, Eeckhoute J, Johnson DS, Bernstein BE, Nusbaum C, Myers RM, Brown M, Li W (2008). Model-based analysis of ChIP-Seq (MACS). Genome Biol.

[34] Kim D, Paggi JM, Park C, Bennett C, Salzberg SL (2019). Graph-based genome alignment and genotyping with HISAT2 and HISAT-genotype. Nat Biotechnol.

[35] Pertea M, Pertea GM, Antonescu CM, Chang TC, Mendell JT, Salzberg SL (2015). StringTie enables improved reconstruction of a transcriptome from RNA-seq reads. Nat Biotechnol.

[36] Sun DQ, Xi YX, Rodriguez B, Park HJ, Tong P, Meong M, Goodell MA, Li W: MOABS: model based analysis of bisulfite sequencing data. Genome Biol. 2014;15(2):R38. 10.1186/gb-2014-15-2-r38 PMC4054608 24565500

[37] Shao W, Wang T (2021). Transcript assembly improves expression quantification of transposable elements in single-cell RNA-seq data. Genome Res.

[38] Dobin A, Davis CA, Schlesinger F, Drenkow J, Zaleski C, Jha S, Batut P, Chaisson M, Gingeras TR (2013). STAR: ultrafast universal RNA-seq aligner. Bioinformatics.

[39] Niknafs YS, Pandian B, Iyer HK, Chinnaiyan AM, Iyer MK (2017). TACO produces robust multisample transcriptome assemblies from RNA-seq. Nat Methods.

[40] Karolchik D, Hinrichs AS, Furey TS, Roskin KM, Sugnet CW, Haussler D, Kent WJ (2004). The UCSC Table Browser data retrieval tool. Nucleic Acids Res.

[41] da Huang W, Sherman BT, Lempicki RA (2009). Systematic and integrative analysis of large gene lists using DAVID bioinformatics resources. Nat Protoc.

[42] Pedregosa F, Varoquaux G, Gramfort A, Michel V, Thirion B, Grisel O, Blondel M, Prettenhofer P, Weiss R, Dubourg V (2011). Scikit-learn: Machine Learning in Python. J Mach Learn Res.

[43] Virtanen P, Gommers R, Oliphant TE, Haberland M, Reddy T, Cournapeau D, Burovski E, Peterson P, Weckesser W, Bright J (2020). SciPy 1.0: fundamental algorithms for scientific computing in Python. Nat Methods.

