# Supplementary material for: Characterization of H3K9me3 and DNA methylation co-marked CpG-rich regions during mouse development
Source: BMC Genomics. 2023 Nov 3;24:663. doi: 10.1186/s12864-023-09758-8 (PMC10623782; doi:10.1186/s12864-023-09758-8)
Supplement: Supplementary file 1 — Additional file 1: Supplementary Figure S1. CHMs are one of the most stable forms at CpG-rich regions during mouse development. Supplementary Figure S2. Environments of CHMs in compartment A and B. Supplementary Figure S3. Potential functions of CHMs in compartment A and B. Supplementary Figure S4. Enrichment of universal CHMs in repeats. Supplementary Figure S5. Potential formation mechanisms of universal CHMs. [file 12864_2023_9758_MOESM1_ESM.pdf]

Fig S1

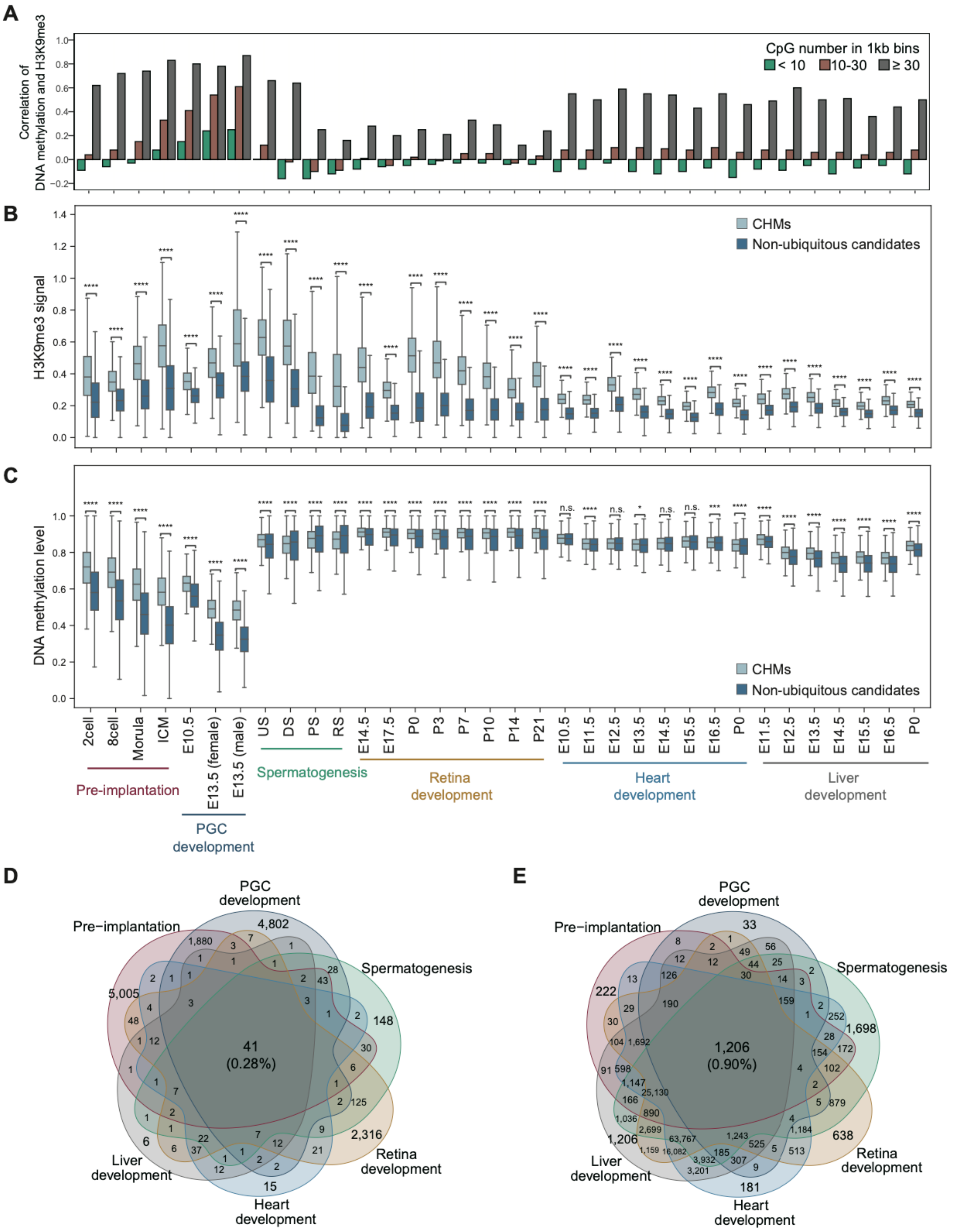

Supplementary Figure S1. CHMs are one of the most stable forms at CpG-rich regions during mouse development.

(**A**) Bar plot showing Pearson's correlation between H3K9me3 signals and DNA methylation levels at different developmental

processes. Three groups of 1-kb bins with different CpG site numbers are shown. (**B-C**) Box plots comparing H3K9me3 signals (**B**) and DNA methylation levels (**C**) at CHMs and non-ubiquitous candidates in corresponding developmental processes. Significance between CHMs and non-ubiquitous candidates was evaluated by a two-sided Wilcoxon rank sum test, ****: *p*-value < 0.0001, ***: *p*-value < 0.001, *: *p*-value < 0.1, n.s.: not significant. The center lines mark the median, the box limits indicate the 25th and 75th percentiles, and the whiskers extend to 1.5× the interquartile range from the 25th and 75th percentiles. (**D-E**) Venn diagram showing the overlap of CH-nonMs (**D**) and CM-nonHs (**E**) in 6 developmental processes.

Fig S2

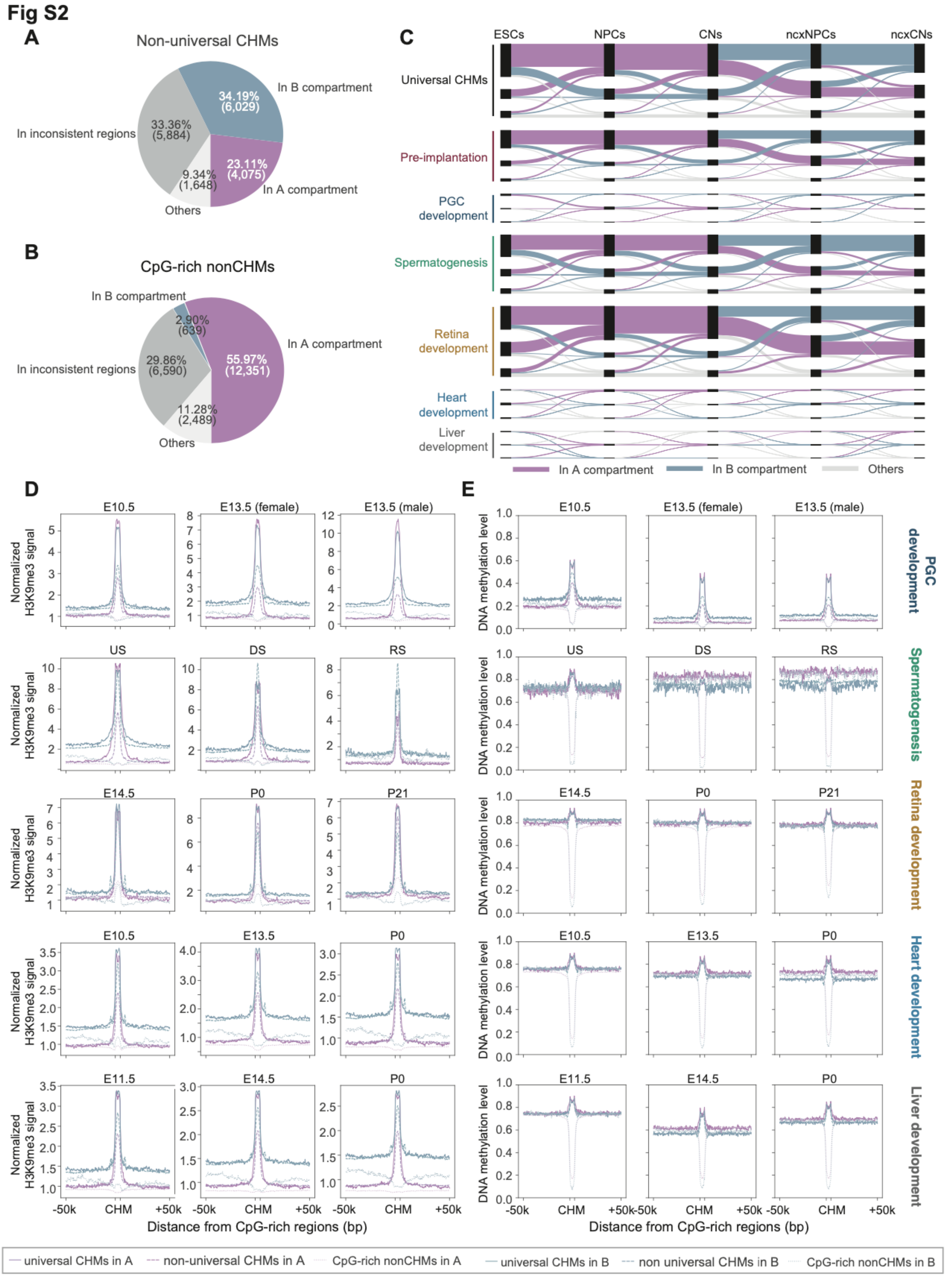

.2

.3 **Supplementary Figure S2. Environments of CHMs in compartment A and B.** (**A-B**) Pie plot showing non-universal

CHMs (**A**) and CpG-rich nonCHMs (**B**) overlapping with compartment A and B. (**C**) Alluvial diagram depicting the numbers of different types of CHMs in inconsistent compartments A (pink), B (blue) and other regions (grey) across five cell types. From Left to Right: ESCs, NPCs, CNs, ncxNPCs, ncxCNs. The height of bars reflect the numbers of regions in corresponding compartments, and the connections between the bars represent regions shared between compartments in different cell types. (**D-E**) Line plots showing H3K9me3 signals (**D**) and DNA methylation levels (**E**) surrounding universal CHMs (± 50 kb, solid), non-universal CHMs (±50 kb, dashed) and CpG-rich nonCHMs (±50 kb, dotted) in compartment A (pink) and B (blue) in the other 5 developmental processes.

Fig S3

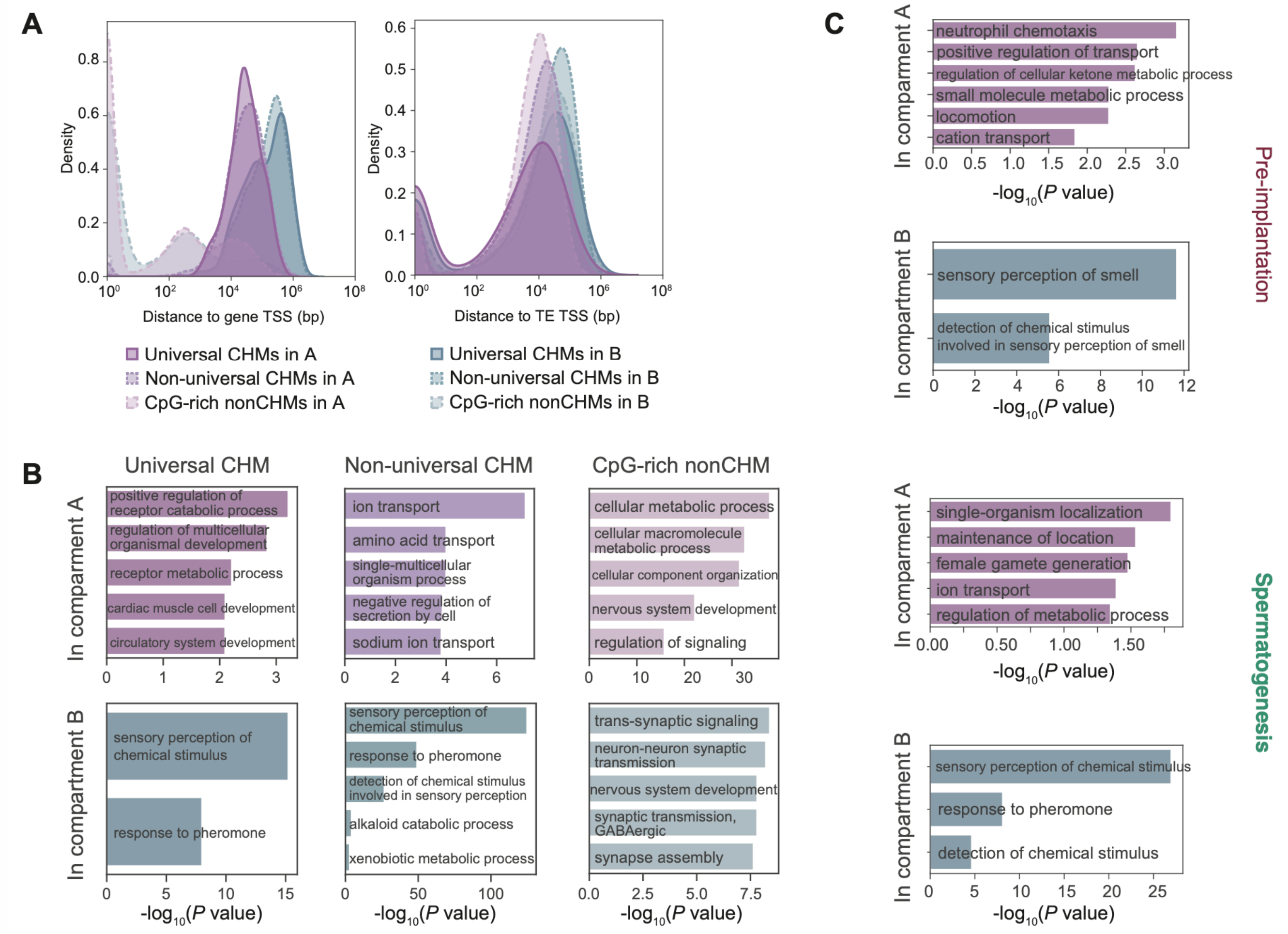

Supplementary Figure S3. Potential functions of CHMs in compartment A and B. (**A**) Density plots showing the distribution of the distance from universal CHMs (dark, solid border), non-universal CHMs (mid-tone, dashed) and CpG-rich nonCHMs (light, unevenly dashed) in compartment A (pink) and B (blue) to genes (top) and TEs (bottom). (**B**) Bar plot showing Gene Ontology (GO) analysis of potential target genes of universal CHMs, non-universal CHMs and CpG-rich nonCHMs in compartment A (left) and B (right). The *p*-values were calculated based on a one-sided Fisher's exact test. (**C**) Bar plot showing Gene Ontology (GO) analysis of potential target genes of pre-impantation-specific CHMs (upper), spermatogenesis-specific CHMs (lower) in compartment A (pink) and B (blue). The *p*-values were calculated based on a one-sided Fisher's exact test. Due to the limited number of CHMs of each process-specific CHM classes, we could only obtain significant GO terms enriched in pre-implantation-specific CHMs and PGC development CHMs.

Fig S4

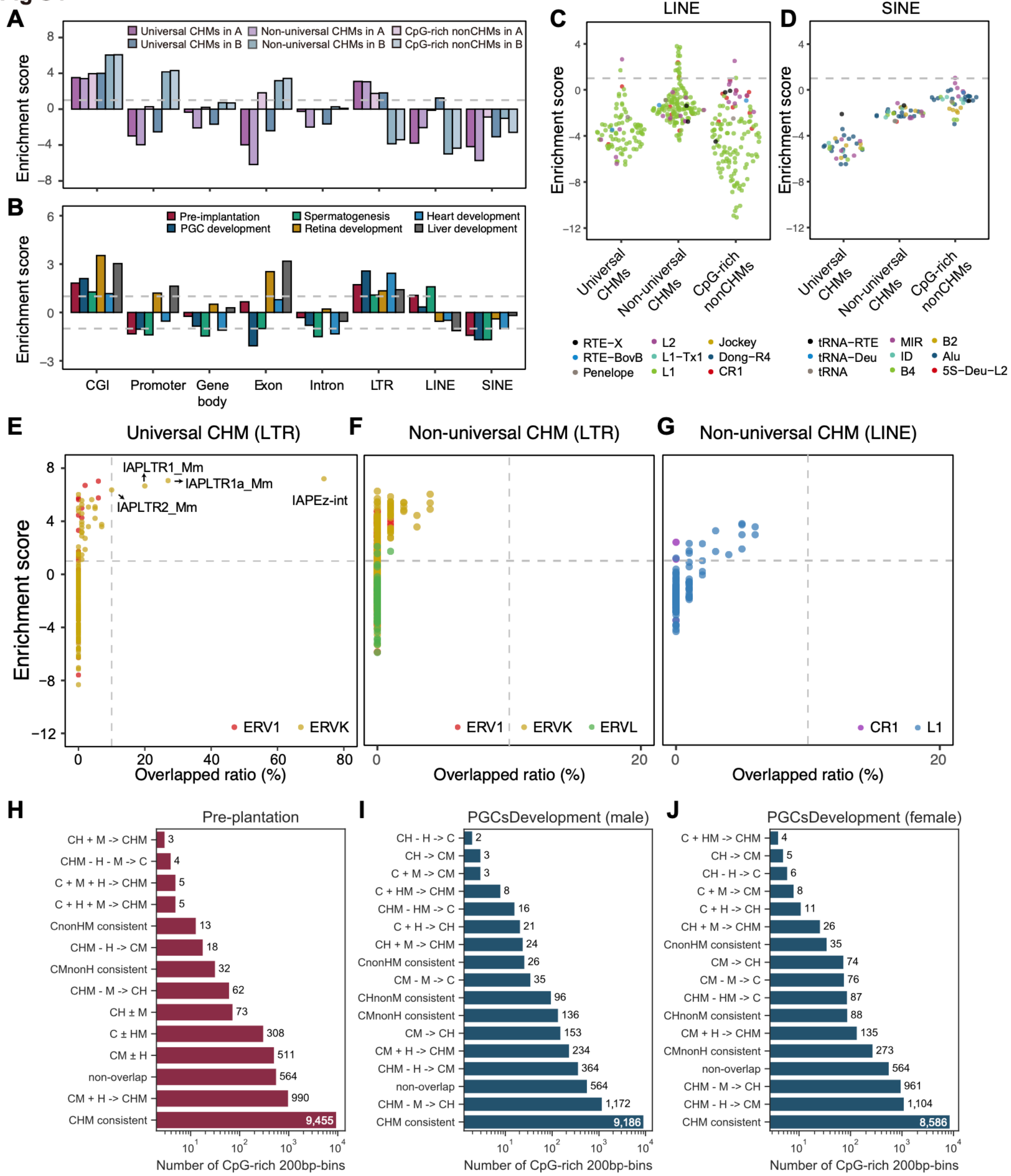

Supplementary Figure S4. Enrichment of universal CHMs in repeats. (**A**) Bar plot showing the enrichment of universal CHMs (dark), non-uninversal CHMs (mid-tone) and CpG-rich nonCHMs (light) in different genomic regions in compartment A (pink) and B (blue). The enrichment score represents the $\log_2$-transformed observed overlapping length/the expected overlapping length ratio. (**B**) Bar plot showing the enrichment of process-specific CHMs in different genomic regions. The

enrichment score represents the log_2-transformed observed overlapping length/the expected overlapping length ratio. (**C-D**) Sina plot showing the enrichment score of universal CHMs, non-universal CHMs and CpG-rich nonCHMs in the LINE (**C**) and SINE (**D**) subfamilies. (**E-G**) Scatter plot showing the enrichment score (y-axis) and overlap ratio (x-axis) of universal CHMs in ERV1 and ERVK from LTR family (**E**), ERV1, non-universal CHMs in ERVK and ERVL from LTR family (**F**), and non-universal CHMs in CR1 and L1 from LINE family (**G**). (**H-J**) Bar plots showing the features of CpG-rich 200bp bins intersecting with IAPEz-int during pre-implantation in the development order of 2-cell, 8-cell, morula and ICM (**H**), and PGC development in the order of E10.5 to E13.5 male (**I**) and E10.5 to E13.5 female (**J**). CpG-rich 200bp bins are defined as genomic bins, each 200 bp in length, containing more than 6 CpGs. "+": gain; "-": loss. "M": 5mC; "H": H3K9men3.

Fig S5

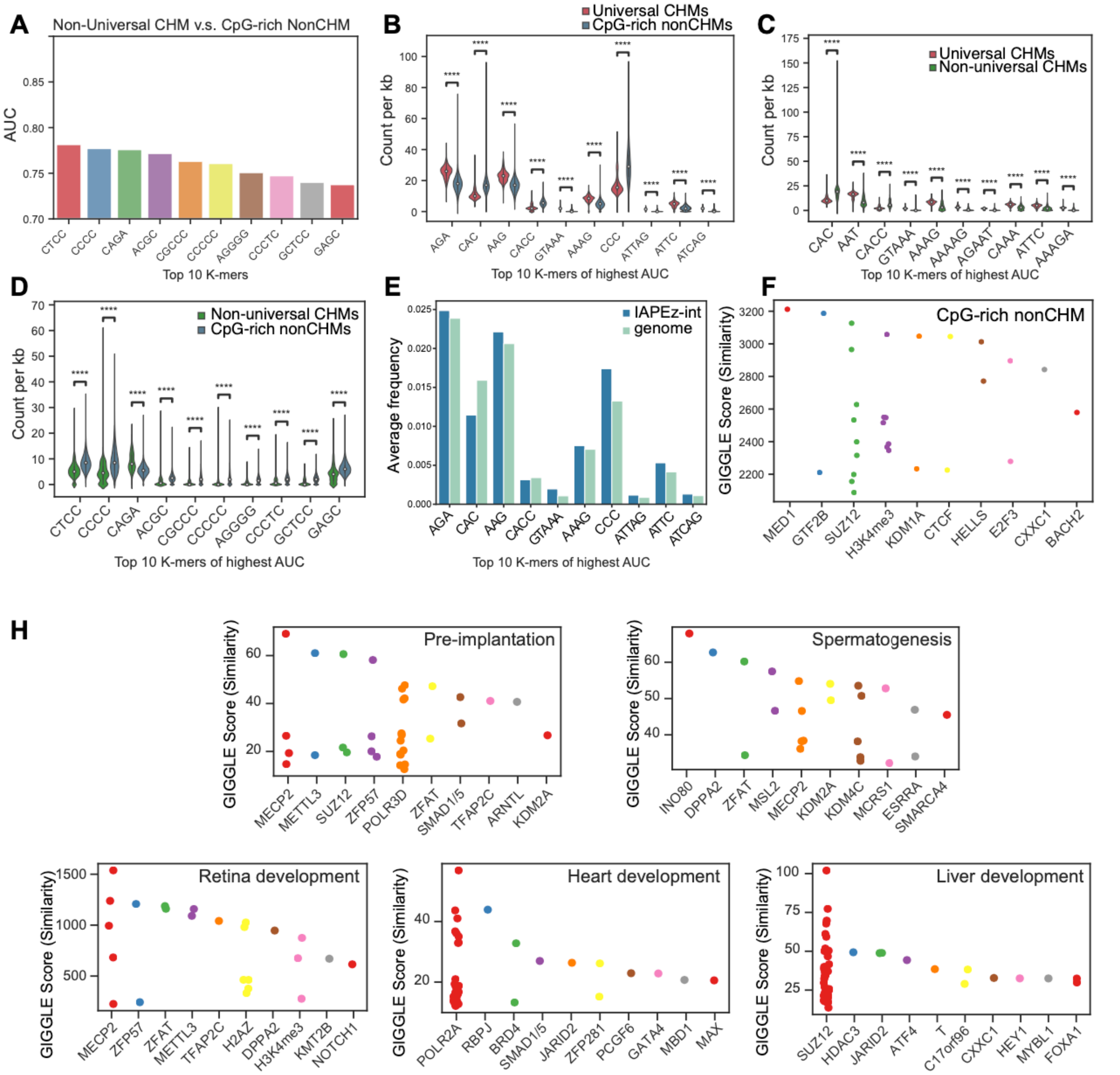

Supplementary Figure S5. Potential formation mechanisms of universal CHMs. (**A**) Bar plot showing the AUC of top 10 k-mers with the best performance in distinguishing non-universal CHMs from CpG-rich nonCHMs. (**B-D**) Violin plot showing the frequency of the top 10 k-mers bestly distinguished universal CHM from CpG-rich nonCHMs (**B**), universal CHMs from universal CHMs from non-universal CHMs (**C**), and non-universal CHMs from CpG-rich nonCHMs (**D**). The frequencies of 1-kb bins from the same universal CHMs were averaged to one number. (**E**) Bar plot showing the average present frequency of the top 10 k-mers in IAPEz-int, IAPLTR1a_Mm and IAPLTR1_Mm. Regions of IAPEz-int, IAPLTR1a_Mm and IAPLTR1_Mm were merged. (**F**) Strip plot showing the top 10 TFs most similar to the CpG-rich

nonCHMs. (**H**) Strip plots showing the top 10 TFs most similar to CpG-rich 1kb-bins of each type of process-specific CHMs. CpG-rich 1kb-bins were defined as genomic 1kb-bins with more than 30 CpGs inside. There was no CpG-rich 1kb-bins of PGC development-specific CHMs.
